# Hereditary ovarian cancer in women with African ancestry: a scoping review

**DOI:** 10.1007/s10689-026-00530-x

**Published:** 2026-01-31

**Authors:** Bianca Rossouw, Monica Araujo, Amanda Krause, Fiona Baine-Savanhu

**Affiliations:** Division of Human Genetics, National Health Laboratory Service and Faculty of Health Sciences, School of Pathology, University of the Witwatersrand, Johannesburg, South Africa

**Keywords:** African population, Germline variants, Hereditary cancer syndromes, Oncology, Ovarian cancer

## Abstract

This scoping review explored genetic variants associated with hereditary ovarian cancer in women of African ancestry. Around 20% of ovarian cancers are hereditary, with *BRCA1* and *BRCA2* variants accounting for 20–55% of these cases, while other implicated genes include *BRIP1, ATM, RAD51C, RAD51D*, and the Lynch syndrome genes. The genetic basis of ovarian cancer in women of African ancestry, however, remains poorly understood. Accurate diagnosis of hereditary cancer syndromes is crucial given their personal and familial implications, informing patient management, surveillance, and cascade testing for at-risk relatives. Eligible studies included women of African ancestry with confirmed primary ovarian, peritoneal, or fallopian tube cancer who underwent germline genetic testing for hereditary cancer syndromes. Studies based solely on somatic testing or genome-wide association approaches were excluded. A comprehensive search of PubMed, Scopus, Web of Science, Google Scholar, and ProQuest Dissertations and Theses Global was conducted. Two reviewers independently screened citations and extracted data using a standardized form, and results were summarised narratively and in tables according to JBI scoping review guidelines. Thirty studies were included, primarily involving African American and North African participants. Only four focused specifically on hereditary ovarian cancer; the remainder primarily examined breast cancer cohorts with some ovarian cancer cases. Across these studies, 75 pathogenic variants were identified in 110 families, with 92% in *BRCA1* or *BRCA2*. Research on hereditary ovarian cancer in women with African ancestry remains extremely limited, highlighting the urgent need for broader next-generation sequencing studies to inform clinical care.

**Supplementary Information:**

The online version contains supplementary material available at 10.1007/s10689-026-00530-x.

## Introduction

Ovarian cancer refers broadly to cancers that begin in the cells of the ovary, fallopian tubes, or peritoneum (the serous membrane that covers the uterus, bladder, rectum, ovaries, and fallopian tubes) [1]. An earlier diagnosis has a better prognosis, but globally less than 20% of women with ovarian cancer are diagnosed with early-stage disease [2]. Clinical symptoms of early disease are usually non-specific, and patients are often diagnosed when the cancer is at an advanced stage [1]. As a result, ovarian cancer is associated with a poor prognosis and survival rate. In South Africa, the 5-year survival rate amongst women with advanced-stage ovarian cancer is only 15–20% despite aggressive treatment [3]. According to the South African National Cancer Registry (SANCR), the number of newly reported cases of ovarian cancer in 2016 was 661, of which 225 (34%) were in African females [4]. Based on available data, the International Agency for Research on Cancer (IARC) predicts that by 2040 the number of cases of ovarian cancer in Africa, driven by expected population growth, will increase by 87%, more than anywhere else in the world [5].

Over 20% of patients with European ancestry and ovarian cancer will have an inherited cancer syndrome with a germline disease-causing variant in one of several genes [6, 7]. A woman’s lifetime risk of developing ovarian cancer is 1–2%, but the presence of a germline disease-causing variant in an ovarian cancer-associated gene significantly increases that risk. Based on current knowledge, the most common cause of inherited ovarian cancer is Hereditary Breast and Ovarian Cancer Syndrome (HBOCS) caused by mutations in either the Breast Cancer gene 1 (*BRCA1*) or Breast Cancer gene 2 (*BRCA2*) [8]. If a woman harbours a mutation in *BRCA1* or *BRCA2* the lifetime risk of developing ovarian cancer increases to as high as 44% [8].

Whilst HBOCS accounts for approximately 20–55% of all inherited ovarian cancer syndromes, several other genes have been implicated. These include Checkpoint Kinase 2 (*CHEK2*), BRCA1 Interacting Protein C-Terminal Helicase 1 (*BRIP1*), Ataxia-Telangiectasia Mutated (*ATM*), RAD51 Paralog C (*RAD51C*), RAD51 Paralog D (*RAD51D*), and MutS Homolog 6 (*MSH6*) Less frequently reported genes include nibrin, (*NBN*), Partner and Localizer of BRCA2 (*PALB2*), Radiation Sensitive 50 (*RAD50*), Meiotic recombination 11 Homolog A (*MRE11A*), MutL homolog 1 (*MLH1*), MutS Homolog 2 (*MSH2*), Postmeiotic Segregation Increased 2 (*PMS2*), BRCA1-associated RING domain 1 (*BARD1*) and Tumor Protein P53 (*TP53*) [9, 10]. Studies from which these genes were derived focused on European cohorts, and it is unclear whether these genes are also strongly associated with ovarian cancer in women with African ancestry and if they are, whether they occur at similar frequencies.

Genetic testing approaches have evolved over time, with the availability of next generation sequencing (NGS) technologies transforming many areas of medicine, including medical oncology. For many years, genetic testing for hereditary breast and/or ovarian cancer involved sequencing the *BRCA1* and *BRCA2* genes only. Genetic testing approaches for hereditary cancer syndromes now typically involve screening a panel of genes which allows clinicians to test for multiple hereditary cancer syndromes in a single assay, increasing the diagnostic yield [11]. Multigene panels typically include high penetrance genes (genes associated with a high risk of developing the condition), and moderate to low penetrance genes (genes associated with low to moderate increased risk of developing the condition). In addition, multigene testing offers a more comprehensive assessment of genes in a cost and time-efficient manner [6]. Multigene panel testing for ovarian cancer has shown increased detection rates of up to 9% for pathogenic variants in *BRCA-*negative patients [12].

In 2016, there was an international drive towards recommending genetic testing for all patients with ovarian cancer [13]. This was due to the release of new drugs called inhibitors of poly-(ADP-ribose) polymerase (PARP inhibitors), which showed improved survival rates in *BRCA-*positive patients with ovarian cancer [14]. Whilst the international recommendation is to test all women with ovarian cancer [15], genetic testing is costly and therefore only patients with specific risk factors tend to be referred for genetic testing in the public healthcare sector in South Africa [16, 17]. These risk factors include ovarian cancer diagnosed under the age of 60 years and a positive family history of associated cancers, such as breast and ovarian. However, the reliability of these factors in identifying all women with inherited ovarian cancer is questionable. Walsh et al. [10] found that more than 30% of patients with ovarian cancer and pathogenic germline variants had no family history of breast or ovarian cancer, and more than 35% of them were 60 years or older at diagnosis. It is therefore important to investigate the genetic basis and risk factors associated with inherited cancer syndromes in individuals with African ancestry, as international recommendations and guidelines may not be appropriate.

A systematic literature review by Rotimi et al*.* [18] found that only 0.016% of cancer genomics publications globally were from Africa. Publications on breast, colorectal, liver and blood cancers represent most of the available cancer-related literature in Africa. The most common genes reported on in African publications include *BRCA1*, *BRCA2* and *TP53*. With precision medicine being an aim for the future, building an African knowledge base is essential to provide patients with optimal care and management. As general screening methods for ovarian cancer are poor, genetic testing plays a role in identifying high risk patients and may contribute to reducing the burden of disease globally. Understanding the genetic aetiology of ovarian cancer in women with African ancestry, a previously overlooked population group, should therefore be a primary research goal.

A preliminary search of PubMed, the Cochrane Database of Systematic Reviews, *JBI Evidence Synthesis,* and Open Science Framework was conducted and no current or underway systematic reviews or scoping reviews on hereditary ovarian cancers in women with African ancestry were identified.

The objective of this scoping review was to identify and summarise the existing literature on hereditary ovarian cancer in women with African ancestry. Information regarding the scope of genetic testing offered and the genetic variants identified in these patients was extracted. The findings of this scoping review have identified important knowledge gaps in this area which will facilitate further research and development.

## Methods

### Review question

What genes have been associated with hereditary ovarian cancer in women with African ancestry?

### Inclusion and exclusion criteria

The genetic aetiology of ovarian cancer has been widely reported in patients with European ancestry. The focus of this review was to identify studies that included women with African ancestry and ovarian cancer, or a family history of ovarian cancer, who underwent germline genetic testing. Studies that involved patients who self-identified as African American, were included. Studies conducted in any African country were included, however, if the study only included patients with European ancestry, it was excluded. Caribbean populations were excluded due to the presence of complex admixture and the possibility of introducing population stratification bias. This scoping review considered analytical observational studies, including prospective and retrospective cohort studies, analytical cross-sectional studies, and descriptive observational study designs including case series, individual case reports and descriptive cross-sectional studies. Systematic reviews were excluded. The literature search included published and unpublished gray literature (e.g. dissertations, theses, conference proceedings). Case–control studies, meta-analyses, and randomized controlled trial studies were excluded as these are not relevant to the research question.

This review was conducted in accordance with the JBI methodology for scoping reviews [19, 20] and PRISMA-ScR guidelines were followed [21]. The review title has been registered on Open Science Framework.

### Search strategy

A three-step search strategy was utilized in this review. An initial limited search of PubMed was undertaken to identify articles on the topic. The text words contained in the titles and abstracts of relevant articles, and the index terms used to describe the articles were used to develop a full search strategy for all databases included. The search strategy, including all identified keywords and index terms, was adapted for each included database. Keywords included terms used to describe ovarian cancer, genetic testing, and countries/populations of interest. All countries and populations of interest were included in one search as a separate Boolean term in the search string.

Only studies published in English were included. No date range was applied. Two reviewers (BR and FB) piloted the search strategy for PubMed. One reviewer (BR) performed the search across all databases. An example of the full search strategy used for PubMed is provided in online resource 1.

The databases searched include PubMed, Scopus, and Web of Science. The search for gray literature included Google Scholar and ProQuest Dissertations and Theses Global.

### Screening and selection

Following the search, all identified citations were collated and uploaded into Mendeley Reference Manager version 2.100.0 2023 and duplicates removed. Titles and abstracts were screened by two independent reviewers (BR and MA) for assessment against the inclusion criteria for the review. Potentially relevant sources were retrieved in full and their citation details imported into the JBI System for the Unified Management, Assessment and Review of Information (JBI SUMARI) (JBI, Adelaide, Australia)[22]. The full text of selected citations was assessed in detail against the inclusion criteria by two independent reviewers (BR and MA). The reference lists of eligible studies were then scanned for further articles. Any disagreements that arose between the reviewers at each stage of the selection process were resolved through discussion.

### Data extraction

Data was extracted from all included articles (BR) using a data extraction tool developed by the authors. The data extracted included specific details about the patients, whether any risk factors were considered before genetic testing was offered, the type of genetic testing done, genetic test results, and tumour histology or disease characteristics where available.

## Results

### Study inclusion

A total of 1095 papers were identified using the search strategy. After title and abstract screening, 923 papers were excluded due to ineligibility. Three papers could not be retrieved for full-text review and a further 143 papers were excluded after full-text review. The main reasons for excluding studies included ineligible phenomena of interest studied, no patients with African ancestry included in the study, or demographics of patients not stated. Many papers were excluded due to insufficient information. This mainly involved papers where patient characteristics (e.g. ethnicity and/or type of cancer) were not linked to reported genetic test results. Four systematic review articles were excluded from the study [23–26]. All relevant original articles from these systematic reviews were included.

**Fig. 1:**
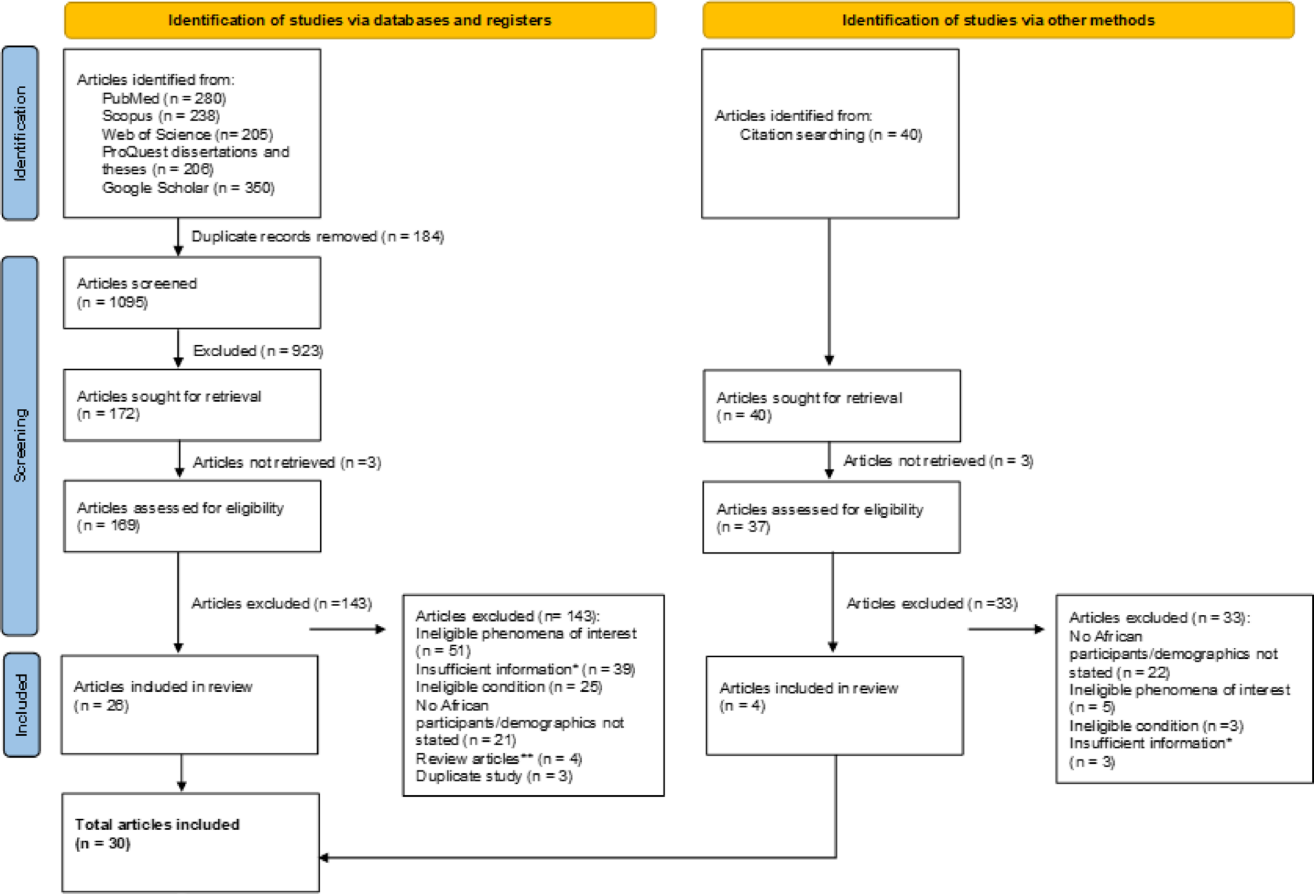
PRISMA 2020 flow diagram illustrating how many articles were included/excluded at each stage of the screening process. 1095 articles were retrieved using the search strategy. After screening, 30 studies met the criteria and were included in the scoping review. *This refers to papers where patients with African ancestry, ovarian cancer, and genetic test results are reported, however, the patient characteristics are not linked to genetic test results. **All relevant original articles in these reviews are included in this scoping review

The reference lists of all included papers were also screened for any relevant papers; this screen yielded an additional four papers for inclusion. Figure 1 shows the PRISMA flow diagram.

### Characteristics of studies

**Fig. 2:**
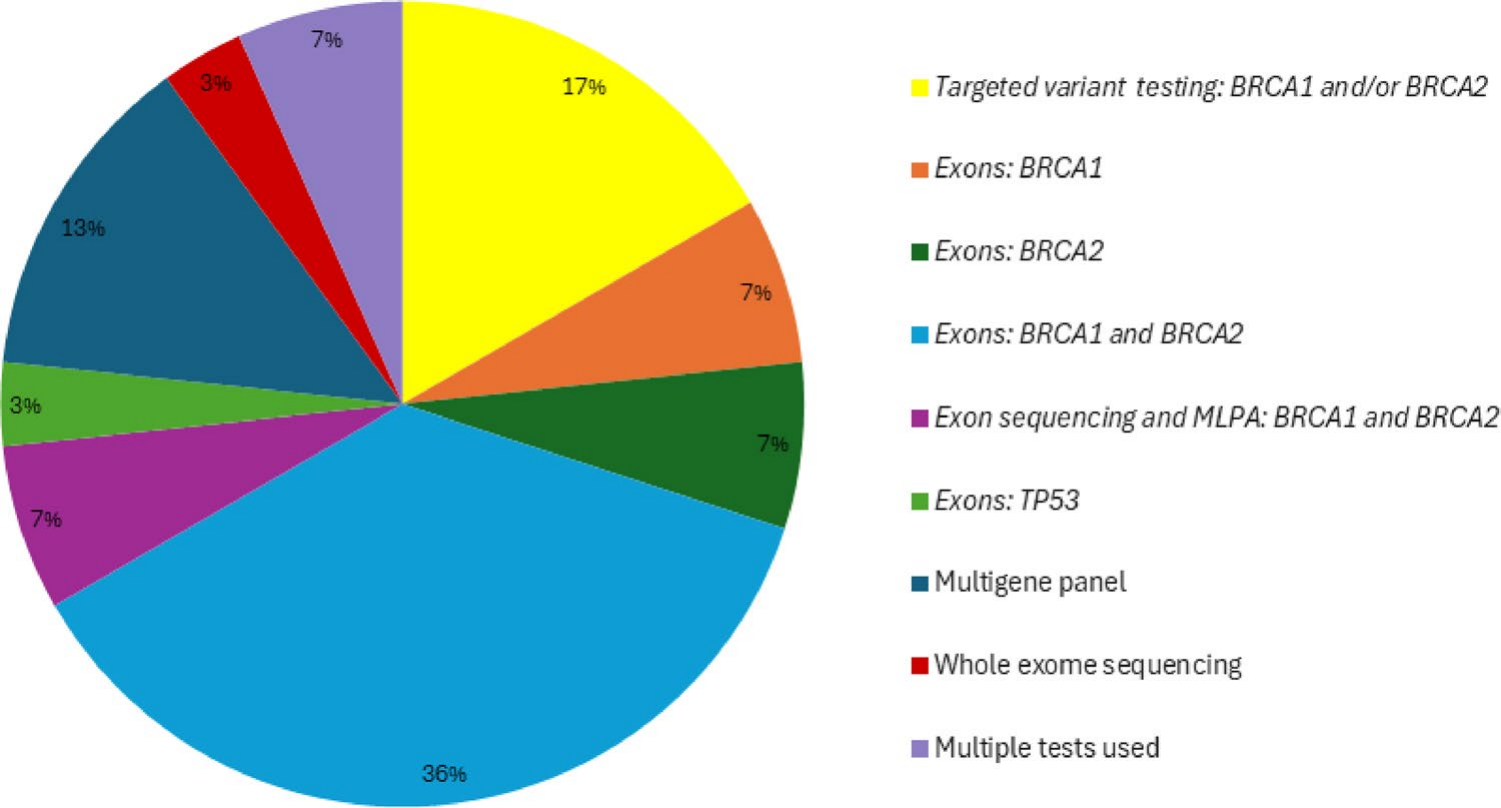
Genetic testing approaches used. Most of the studies used limited genetic testing approaches, with a focus on *BRCA1* and *BRCA2* analysis. Targeted variant testing used PCR or Sanger sequencing approaches, exon sequencing mainly used Sanger sequencing approaches, and gene panel testing and whole exome sequencing used next-generation sequencing approaches. Some studies used multiple tests, e.g. targeted variant testing followed by full-gene exon sequencing

Thirty studies were included in this scoping review. The supplementary information (online resource 2) contains a table of study characteristics. The majority were performed in the United States of America (USA) (14/30) and North Africa (13/30). Two studies were conducted in South Africa, and one study was conducted in Europe (Table 1). Patient cohorts were therefore mainly African American or North African (Tunisian, Moroccan, or Algerian). Very few genetic studies including ovarian cancer patients have been performed on the African continent, particularly in Sub-Saharan Africa (2/30). Most of the studies focused on *BRCA1* and *BRCA2* gene testing (Fig. 2).

**Table 1:** Distribution of included studies

Study origin	Number of papers	Cohort included (n = number of papers)
United States of America*	14	African American (n = 14) West African (n = 1)**
North Africa	13	Tunisian (n-6) Moroccan (n = 5) Algerian (n = 2)
South Africa	2	South African (n = 1) Zimbabwean (n = 1)
Europe	1	Moroccan (n = 1)
	Total = 30	

*Studies in the United States of America included patients from the states of California, Florida, Georgia, Illinois, New York and Washington

**One paper included both African American and West African cohorts

### Genetic testing outcomes in ovarian cancer patients with African ancestry

Twenty-five studies included patients who had been offered genetic testing based on meeting high-risk criteria of having a hereditary cancer syndrome. The criteria applied varied across studies, but most considered a young age of onset and a family history of breast and/or ovarian cancer. Only four studies [27–30] focused solely on hereditary ovarian cancer, irrespective of age of diagnosis and family history. One study included breast and/or ovarian cancer patients and did not include any other testing criteria. The majority (26/30) of studies involved hereditary breast cancer cohorts.

Fourteen studies did not report patient characteristics for the complete study cohort and only provided information on patients/families who tested positive for a disease-causing variant. Information provided was also inconsistent between studies. Only four studies included tumour histology information [27, 30–32]; however, this was not always linked to the genetic test results and so this was not included in the analysis. The total number of individuals with ovarian cancer in each study cohort was not always clear and therefore an accurate diagnostic yield could not be calculated. Based on the information extracted from the included studies, there were at least 352 probands with African ancestry diagnosed with ovarian cancer. Of these probands, 59/352 (16.8%) had a disease-causing variant identified. There were 119 probands who had a different primary cancer (most often breast cancer) and had a family history of ovarian cancer (family history varied from one affected family member to multiple affected family members and different degrees of relatedness). A total of 51/119 (42.9%) non-ovarian cancer probands with a family history of ovarian cancer had a disease-causing variant identified. There were 132 ovarian cancer cases documented (59 probands; 73 family members) in 110 families that had a disease-causing variant identified. Of these individuals, 64/132 (48.5%) were confirmed to carry the disease-causing variant. The rest of the affected family members were assumed carriers but were not tested. Only 7/59 (11.9%) ovarian cancer patients with a confirmed disease-causing variant had a documented family history of ovarian cancer.

**Fig. 3:**
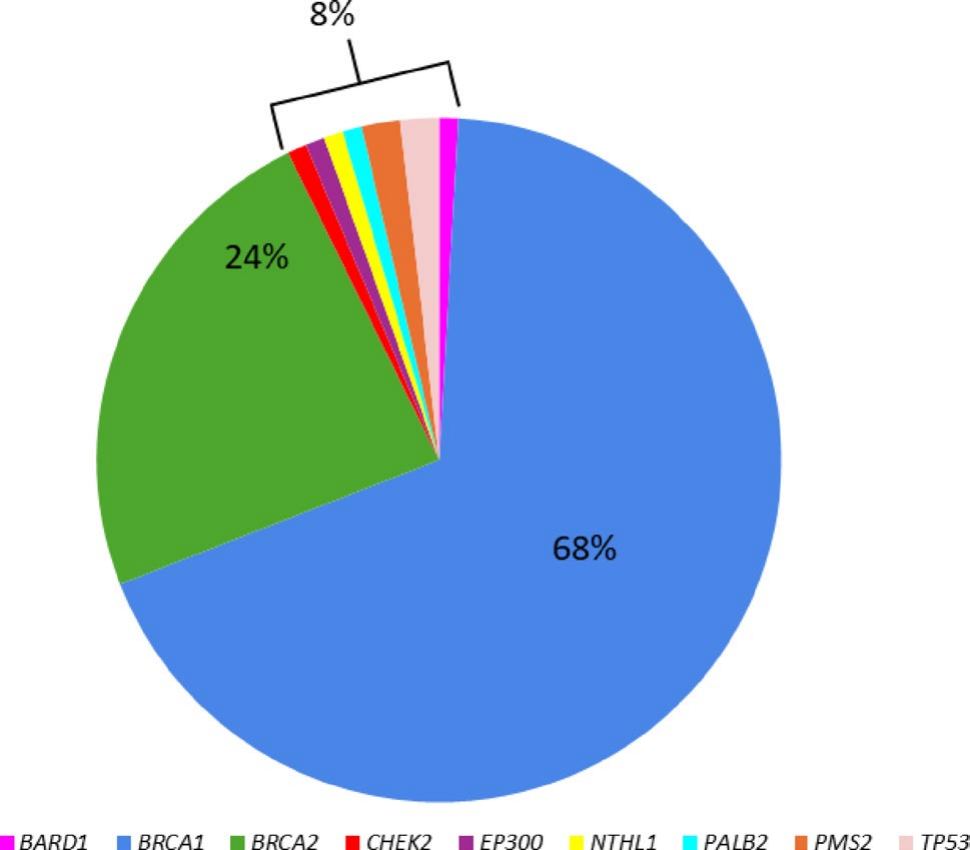
Genes associated with hereditary ovarian cancer in women with African ancestry. *BRCA1* (68%) and *BRCA2* (24%) were the genes most frequently associated with hereditary ovarian cancer in the included studies

Disease-causing variants in *BRCA1* and *BRCA2* account for 92% of the variants reported in the included studies. Other reported genes include *BARD1, CHEK2, EP300* lysine acetyltransferase (*EP300*), nth like DNA glycosylase 1 (*NTHL1*)*, PALB2, PMS2 and TP53* (Fig. 3). BenAyed-Guerfali et al. [33] performed whole exome sequencing (WES) on *BRCA*-negative patients from Tunisian families with a high risk of having a hereditary breast and/or ovarian cancer syndrome and reported a likely pathogenic variant in *EP300* in a proband with breast cancer. The proband had a family history of ovarian cancer, however, the affected family members were not tested for this variant. Germline variants in this gene have not been associated with hereditary cancer syndromes previously and the association of *EP300* with HBOC requires further investigation.

A summary table of the disease-causing variants reported in ovarian cancer families can be found in the supplementary information (online resource 3). Each variant was only counted once per family, even if multiple family members carried the variant. In total, 75 different variants were reported in 110 African ovarian cancer families. Some variants in the *BRCA1* and *BRCA2* genes were identified in more than one family and reported in more than one study. These variants are summarised in Table 2. Whilst most variants were unique, it is not unexpected to identify recurrent variants within a population group.

**Table 2:** *BRCA1/2* variants identified in more than one family

Variant	Number of families	Country	References
*BRCA2* c.1310_1313delAAGA (p.Lys437Ilefs*22)	5	Morrocco; Algeria	[34–36]
*BRCA1* c.943_944ins10 (p.Ala315Tyrfs*289)	5	USA	[37–39]
*BRCA1* c.5309G > T (p.Gly1770Cys)	4	Morrocco	[36, 40]
*BRCA1*c.5030_5033delCTAA (p.Pro1677Glnfs*7)	3	Tunisia	[41]
*BRCA1* c.1832del5 (p.Thr611Serfs*3)	3	USA	[42, 43]
	Total = 20		

## Discussion

### Limited studies available that include African cohorts

To the best of our knowledge, this is the first scoping review focusing on the genetic aetiology of ovarian cancer in women with African ancestry. Only 30 publications met the inclusion criteria and most of these (14/30) were conducted in the USA, on African American women. Patients with African ancestry represented a small proportion of the patient cohorts across these studies. In studies performed in South Africa, cohorts were disproportionately or exclusively comprised of patients with European ancestry. Studies that only included patients with European ancestry were excluded. Of the studies performed on the African continent (Table 1) majority were based in North Africa (13/15). Based on the findings of this review, ovarian cancer has not been prioritised in research, with most relevant cases being identified through breast cancer research cohorts. Caribbean populations, comprising varying proportions of African, European, Asian and Indigenous American ancestry, were excluded from this review. Complex admixture such as this can introduce population stratification bias and may confound analyses. Had these studies been included, additional variants may have been captured but attributing those as African specific variants would be difficult. One study performed in Europe was included. There are likely other published European studies that include individuals of African ancestry in their cohorts, however, if the study did not clearly specify that individuals of African ancestry were included in the study, it would have been excluded from this review.

There is a paucity of ovarian cancer studies in all sub-Saharan Africa. A noteworthy discovery is the absence of published data on germline genetic testing results in ovarian cancer patients with African ancestry in South Africa, despite South Africa's established clinical genetics infrastructure [44]. Possible explanations include the limited focus on ovarian cancer in research recruitment, poor access to clinical services for affected individuals, or the publication of data without adequate reporting of ethnicity and patient characteristics. This suggests broader systemic barriers in health care access, referral processes, and a lack of research participation among African ancestry populations. Addressing these barriers requires targeted efforts to increase awareness, streamline referral pathways, and promote equitable inclusion in research.

Africa is a large and genetically diverse continent which makes it critical for genetic studies to include cohorts from across the continent. North Africans are genetically distinct from Sub-Saharan Africans due to a complex interplay of geography, historical migrations, and long-term population isolation and admixture. The Sahara Desert has acted as a partial barrier to gene flow, while North Africa’s proximity to the Middle East and Europe has resulted in greater genetic exchange with those regions [45, 46]. African Americans are primarily of West African origin, a conclusion supported by the transatlantic slave trade, genetic studies, and cultural connections [47, 48]. This diversity supports the need of larger and directed studies across Africa due to the difficulty of extrapolating genetic data from one area to another.

### Patients with African ancestry have limited access to broad-based genetic testing approaches

The review highlights the overreliance on *BRCA1* and *BRCA2* testing (Fig. 2). While these genes are central to hereditary ovarian cancer globally, there are other prevalent high and moderate risk genes associated with ovarian cancer, such as *BRIP1, ATM, PALB2*, *RAD51C, RAD51D*, *CHEK2*, *PMS2*, and *TP53* [12, 49, 50]. Research focusing on hereditary breast cancer has dominated the cancer genetics research space in Africa. Furthermore, most studies have focused primarily on *BRCA1* and *BRCA2* with limited broad exploratory studies being performed [18, 51]. This finding is not unexpected, as *BRCA1* and *BRCA2* genetic testing has been available the longest, and there is limited availability of newer technologies using NGS in low resource settings which is reflected in the lack of use in studies. This limits the scope of variant discovery and hampers efforts to develop population-specific genetic testing strategies. As a result, our knowledge of the contribution of genes other than *BRCA1* and *BRCA2* is limited.

Directing testing appropriately is critical. In a study looking at South African ovarian cancer patients with African ancestry [30], genetic testing involved testing patients for the Ashkenazi Jewish founder mutations in *BRCA1* and *BRCA2*. It is therefore not surprising that no mutations were identified (online resource 2). This is an important example of how the wrong testing approach was used, and had broader testing been implemented, patients with an underlying hereditary cancer syndrome could have been identified. Based on the testing that was done in this study, you cannot exclude a diagnosis of a hereditary cancer syndrome in any of these patients as the testing done was inappropriate and not relevant to this population group. Given the unique genetic diversity within African populations, broader testing approaches such as those using NGS panels or exomes are critical for both research and clinical applications. It is evident from other studies that there are clinically significant variants in genes other than *BRCA1* and *BRCA1* associated with ovarian cancer. This motivates the need to expand genetic testing to expand our knowledge on the spectrum of variants and their contributions to hereditary ovarian cancer in patients with African ancestry.

In addition, the lack of diversity in genetic research studies limits the accuracy of variant interpretation, which can lead to identifying a higher proportion of variants of uncertain significance (VUS) in individuals with African ancestry. Expanding NGS testing in this population group not only helps provide more equitable access, but also contributes critical data needed to improve variant classification.

The studies included in this review reported 75 different disease-causing variants in 110 families with African ancestry and ovarian cancer, the vast majority (92%) of which were in *BRCA1* and *BRCA2*. A few variants were seen in multiple families or across different studies (Table 1). While some of these variants may represent founder mutations in these regions, more studies in larger cohorts are required to establish whether these are more likely to be recurrent mutations or population specific founder mutations. These findings reinforce the need for well-designed studies that assess the prevalence and penetrance of these variants in diverse African populations. The number of affected individuals with confirmed pathogenic variants also underscores the importance of offering genetic testing in appropriate clinical settings. Among the 352 probands with ovarian cancer, 59 (16.8%) were found to have a disease-causing variant. Only 7/59 (11.9%) ovarian cancer patients with a confirmed disease-causing variant had a documented family history of ovarian cancer. Using family history as a criterion for assessing hereditary cancer syndrome risk may therefore not always be appropriate in this population group. The absence of a family history of cancer does not exclude an underlying hereditary cancer syndrome, and using this as a criterion for genetic testing will result in affected families going undetected. In addition, many cases of ovarian cancer may go undetected due to vague symptoms, poor detection in early stages, and advanced progression. Other criteria that are generally considered for genetic testing for hereditary ovarian cancer include a young age of onset (ovarian cancer diagnosed younger than 60 years of age), tumour histology (high-grade serous carcinoma), bilateral disease, and diagnosis of multiple primary tumours (e.g. breast and ovarian cancer).

### Incomplete information and inconsistent reporting noted across studies

A major barrier to drawing meaningful clinical insights from this dataset was the inconsistent reporting. A limitation noted across many studies was the poor linkage of patient characteristics to genetic test results. Forty-two publications were excluded due to incomplete information. While these studies referenced African individuals, ovarian cancer patients, and genetic testing, they failed to clarify whether these variables were connected, for example, whether the African individuals were the ovarian cancer patients, or whether genetic test results were reported for either group. As a result, many publications were excluded, despite containing potentially relevant data.

Reporting inconsistencies are further exacerbated when researchers do not have access to all required clinical data and therefore cannot include these types of analyses as part of their research. Only a handful of studies in this review included information such as tumour histology and age at diagnosis, and even fewer linked this to genetic findings. Many studies did not indicate the total number of ovarian cancer patients included in the cohort and only reported on the patients with a confirmed disease-causing variant. This significantly limits our ability to determine diagnostic yield, assess genotype–phenotype correlations, and create guidelines for genetic testing. Improved reporting of such variables is essential to fully realise the clinical utility of genetic studies.

The inclusion of clinical data is increasingly important when reporting variants in databases such as ClinVar. In addition, databases with rich clinical information, such as National Cancer Registries, should start consistently incorporating genomic data as part of their reporting. Reporting variants without linking the clinical data and patient ancestry limits clinical utility and hinders the translation of research and diagnostic findings into clinical practice. This same issue is highlighted by Lim et al. [52] who created reporting guidelines for precision medicine research to ensure that research findings are reported in a standardised manner to enable implementation into clinical practice. The BePRECISE checklist was created to guide authors when writing their research papers. Guidelines which assist authors in appropriately reporting results from genetic studies will support the translation of clinical research and incorporation of results into clinical guidelines.

As a start, we recommend that all cancer genetic studies should strive to include the following information where possible: patient ethnicity, age at diagnosis, cancer type, and genetic test results. Where relevant, additional details such as tumour histology, treatment regimens, and co-morbidities should also be reported. Crucially, patient characteristics must be explicitly linked to the genetic findings. Without this connection, it is impossible to assess risk factors or testing outcomes in a clinically meaningful way, and it becomes difficult to build on previous knowledge with new studies as the key data are not available.

## Conclusion

This review highlights the limited scope of research on hereditary ovarian cancer, particularly in patients with African ancestry. Limited knowledge exists on the genetic aetiology and epidemiology of ovarian cancer in patients with African ancestry, and this should become a primary research focus. This is required so that diagnostic testing services are appropriately targeted.

The clinical implications of having an underlying hereditary cancer syndrome are significant. Once a diagnosis is made there are immediate and long-term implications for patient care, management, treatment, and family planning. Furthermore, the implications extend beyond the individual, as multiple family members are at risk of being affected and may benefit from predictive testing, cancer surveillance, and early intervention. The identification of a hereditary cancer syndrome can inform the care and prevention strategies for entire family units. Therefore, understanding the genetic aetiology of hereditary ovarian cancer is not only important for optimizing individual treatment pathways, but also for enabling cascade testing and broader public health interventions. Integrating genetic services into oncology care pathways and improving the reach of genetic counselling is essential to ensure patients and their families receive comprehensive, equitable care.

Finally, the limited research output from African institutions reflects a broader issue of inequitable funding and research capacity. Strengthening local research ecosystems through sustainable funding, training programs, biobank development and collaborations will be vital to enable African-led genomic research and improve health equity. Research investigating the genetic aetiology of ovarian cancer in women with African ancestry and the associated risk factors should be a research priority so that appropriate genetic testing guidelines and testing approaches can be implemented in a clinical setting.

## Supplementary Information

Below is the link to the electronic supplementary material.

Supplementary Material 1

Supplementary Material 2

Supplementary Material 3

## Data Availability

The data that supports the findings of this study are available in the supplementary material of this article.
