# Supplementary material for: Hereditary ovarian cancer in women with African ancestry: a scoping review
Source: Fam Cancer. 2026 Jan 31;25(1):16. doi: 10.1007/s10689-026-00530-x (PMC12860814; doi:10.1007/s10689-026-00530-x)
Supplement: Supplementary file 1 — Supplementary Material 1 [file 10689_2026_530_MOESM1_ESM.docx]

**Supplementary: Search strategy used to identify articles on hereditary ovarian cancer in patients with African ancestry**

Search String used in PubMed:

(Africa[All Fields] OR Algeria[All Fields] OR Angola[All Fields] OR Benin[All Fields] OR Botswana[All Fields] OR "Burkina Faso"[All Fields] OR Burundi[All Fields] OR "Cabo Verde"[All Fields] OR Cameroon[All Fields] OR "Central African Republic"[All Fields] OR Chad[All Fields] OR Comoros[All Fields] OR Congo[All Fields] OR "Côte d'Ivoire"[All Fields] OR "Democratic Republic of the Congo"[All Fields] OR Djibouti[All Fields] OR Egypt[All Fields] OR "Equatorial Guinea"[All Fields] OR Eritrea[All Fields] OR Ethiopia[All Fields] OR Gabon[All Fields] OR Gambia[All Fields] OR Ghana[All Fields] OR Guinea[All Fields] OR "Guinea-Bissau"[All Fields] OR Kenya[All Fields] OR Lesotho[All Fields] OR Liberia[All Fields] OR Libya[All Fields] OR Madagascar[All Fields] OR Malawi[All Fields] OR Mali[All Fields] OR Mauritania[All Fields] OR Mauritius[All Fields] OR Morocco[All Fields] OR Mozambique[All Fields] OR Namibia[All Fields] OR Niger[All Fields] OR Nigeria[All Fields] OR Rwanda[All Fields] OR "São Tomé and Príncipe"[All Fields] OR Senegal[All Fields] OR Seychelles[All Fields] OR "Sierra Leone"[All Fields] OR Somalia[All Fields] OR "South Africa"[All Fields] OR "South Sudan"[All Fields] OR Sudan[All Fields] OR Swaziland[All Fields] OR Togo[All Fields] OR Tunisia[All Fields] OR Uganda[All Fields] OR Tanzania[All Fields] OR Zambia[All Fields] OR Zimbabwe[All Fields] OR "Cape Verde"[All Fields] OR "République centrafricaine "[All Fields] OR "Ivory Coast"[All Fields] OR Zaire[All Fields] OR DRC[All Fields] OR black [All Fields] OR Eswatini[All Fields] OR "Sub-Saharan Africa"[All Fields] OR "West Africa"[All Fields] OR "East Africa"[All Fields] OR "North Africa"[All Fields] OR "Southern Africa"[All Fields] OR "African population "[All Fields]) NOT ("African-American "[tw]) AND (genetic [All Fields] OR gene[All Fields] OR germline[All Fields] OR mutation[All Fields] OR hereditary[All Fields] OR inherited[All Fields] OR "hereditary cancer syndrome "[All Fields] OR "inherited cancer syndrome "[All Fields] OR "cancer syndrome "[All Fields] OR familial [All Fields] OR "familial cancer syndrome "[All Fields] OR BRCA1[All Fields] OR BRCA2[All Fields] OR "hereditary breast and ovarian cancer syndrome "[All Fields] OR HBOCS[All Fields] OR "Lynch syndrome "[All Fields] OR "founder mutation "[All Fields] OR "multigene panel "[All Fields] OR "gene panel"[All Fields] OR "exome sequencing "[All Fields] OR "genome sequencing "[All Fields]) NOT (GWAS[All Fields]) NOT (SNP[All Fields]) NOT (somatic[All Fields]) NOT (therapy[tiab]) NOT (pharmacogenomics[tiab]) NOT (therapeutic[tiab]) NOT (treatment[tiab]) NOT (drug[tiab]) NOT (Trial [tiab]) NOT ("case-control"[tiab]) AND ("ovarian cancer "[All Fields] OR "ovary cancer"[All Fields] OR "fallopian tube cancer"[All Fields] OR "peritoneal cancer"[All Fields]) NOT ("clinical trial"[pt] OR "meta-analysis"[pt] OR "randomized controlled trial "[pt]) AND (english[la]) AND (human[All Fields])
