# Supplementary material for: Hereditary ovarian cancer in women with African ancestry: a scoping review
Source: Fam Cancer. 2026 Jan 31;25(1):16. doi: 10.1007/s10689-026-00530-x (PMC12860814; doi:10.1007/s10689-026-00530-x)
Supplement: Supplementary file 2 — Supplementary Material 2 [file 10689_2026_530_MOESM2_ESM.docx]

**Online resource 2**

**Characteristics of studies included for analysis in scoping review on the genetic aetiology of ovarian cancer in patients with African ancestry**

_BC: breast cancer; OC: ovarian cancer; BC/OC: Patient with breast and ovarian cancer; FHx: family history; USA: United States of America_

_Documented variants were identified in unrelated patients/families._

| **Study** | **Country study conducted in** | **Setting/context** | **Genetic Test performed** | **Summary of main results** |
| --- | --- | --- | --- | --- |
| Cherbal F, Bakour R, Adane S, Boualga K, Benais-Pont G, Maillet P. 2010. [34] | Algeria | Analysis of *BRCA1* and *BRCA2* genes in 86 individuals from 70 families from an Algerian cohort with a personal and family history suggestive of genetic predisposition to BC recruited from Anti-Cancer Centre of Blida, the Central Hospital of Algiers, and three private medical clinics | Sequencing and MLPA of *BRCA1* and *BRCA2* | 3 variants identified in *BRCA1:*   - 1 patient with BC/OC - 2 patients with FHx OC   1 variant identified in *BRCA2:*   - 1 patient with FHx OC |
| Mehemmai C, Cherbal F, Hamdi Y, Guedioura A, Benbrahim W, Bakour Rabah. 2019. [53] | Algeria | Investigated the mutation spectrum of *BRCA1* and *BRCA2* in high-risk families from the Aures region (eastern Algeria). Patients collected from cancer registry of anticancer centre of Batna between 2011 and 2015. | Targeted mutation testing for *BRCA1* and *BRCA2* mutations previously identified in Algerian patients  12 patients selected for NGS gene panel (30 genes) or full *BRCA1/BRCA2* sequencing | 3 variants identified in *BRCA1:*   - 1 Patient with BC/OC and FHx OC - 1 patient with OC and FHx OC - 1 patient with FHx OC   3 variants identified in *BRCA2:*   - 1 Patient with BC/OC and FHx OC - 2 patients with FHx OC |
| El Ansari FZ, Jouali F, Marchoudi Nabila, Ghailani NN, Barakat A, Fekkak J. 2020. [31] | Morocco | Analysed *BRCA1/2* variants and copy number variations in 64 selected Moroccan patients, with BC and/or OC and had a strong family history for cancer. Patients referred to referred to ANOUAL laboratory for *BRCA1/2* genetic testing between 2016 and 2020 | *BRCA1* and *BRCA2* sequencing | 4 variants identified in *BRCA1:*   - 1 patient with BC/OC - 2 patients with OC - 1 patient with FHx OC   3 variants identified in *BRCA2:*   - 3 patients with OC |
| **Study** | **Country study conducted in** | **Setting/context** | **Genetic Test performed** | **Summary of main results** |
| Elalaoui SC, Laarabi FZ, Afif Lamiae, Ratbi I, Jaouad Imane Cherkaoui, Sahli M, et al. 2022. [35] | Morocco | Evaluated 163 unrelated Moroccan patients with BC and/or OC recruited from an oncogenetic outpatient clinic | Targeted mutation testing: *BRCA2* c.1310_1313delAAGA mutation at exon 10 and *BRCA1* and *BRCA2* sequencing (coding region and flanking intronic regions) | 7 variants identified in *BRCA1:*   - 2 patients with OC - 1 patient with BC/OC - 4 patients with FHx OC   5 variants identified in BRCA2:   - 2 patients with OC - 1 patient with OC and FHX OC - 2 patients with FHx OC |
| Laarabi FZ, Jaouad IC, Ouldim K, Aboussair N, Jalil A, Gueddari BEKE, et al. 2011. [32] | Morocco | Presymptomatic diagnosis was carried out using DNA genetic testing in 5 healthy Moroccan female individuals from three families with an elevated risk of developing breast cancer. These are the first Moroccan families reported to be affected by breast cancers associated with *BRCA* mutations. | Sequencing *BRCA1* and *BRCA2* (coding regions) | 1 variant identified in *BRCA1:*   - 1 patient with FHx OC |
| Melki R, Melloul M, Aissaoui S, El, Boukhatem N. 2023. [36] | Morocco | Evaluated 184 BC Moroccan patients referred from Hassan II Regional Oncology Centre of Oujda, the Northeastern region of Morocco, for the *BRCA1* and *BRCA2* founder mutations | Targeted testing *BRCA1* c.5309G>T and *BRCA2* c.1310_1313delAAGA | 2 variants identified in *BRCA1:*   - 2 patients with FHx OC   1 variant identified in *BRCA2*:   - 1 patient with FHx OC |
| Tazzite A, Jouhadi H, Nadifi S, Aretini P, Falaschi E, Collavoli A, et al. 2012. [54] | Morocco | Study included 40 Moroccan early onset and familial BC and OC cases selected from patients treated in oncology centre of Ibn Rochd University Hospital of Casablanca | *BRCA1* and *BRCA2* sequencing (coding regions and flanking intronic regions) | 1 variant identified in *BRCA1:*   - 1 patient with FHx BC/OC |
| **Study** | **Country study conducted in** | **Setting/context** | **Genetic Test performed** | **Summary of main results** |
| Troudi W, Uhrhammer N, Romdhane KB, Sibille C, Amor MB, Khodjet El Khil H, et al. 2008. [55] | Tunisia | Investigated *BRCA1* mutations in 32 unrelated Tunisian patients who had at least one first degree relative affected with BC and/or OC at Salah Azaiz Institute of Carcinology | BRCA1 sequencing (coding regions) | 4 variants identified in *BRCA1:*   - 1 patient with BC/OC and FHx OC - 2 patients with BC/OC - 1 patient with FHx BC/OC |
| Ben Ayed-Guerfali D, Ben Kridis-Rejab W, Ammous-Boukhris N, Ayadi W, Charfi S, Khanfir A, et al. 2021. [41] | Tunisia | 134 Tunisian patients with BC and/or OC and high risk of HBOCS recruited between 2016 and 2019 from the department of Medical Oncology of the CHU Habib Bourguiba of Sfax | *BRCA1* and *BRCA2* sequencing (coding regions) | 5 variants identified in BRCA1:   - 4 patients with OC - 1 patient with BC/OC |
| BenAyed-Guerfali D, Kifagi C, BenKridis-Rejeb W, Ammous-Boukhris N, Ayedi Wajdi, Daoud J, et al. 2022. [33] | Tunisia | Exome Sequencing of BRCA-negative patients from Tunisian families with a high risk of HBOC. | Exome sequencing | 1 variant identified in *BRCA1*:   - 1 patient with OC   1 variant identified in *BRCA2:*   - 1 patient with FHx OC   1 variant identified in *EP300*:   - 1 patient with FHx OC |
| Fourati A, Louchez M-M, Fournier Joelle, Rahal K, El May M-V, El May A, et al. 2014. [56] | Tunisia | Evaluated 66 Tunisian patients, with family history of BC and/or OC, recruited from Salah Azaiz Cancer Institute | Sanger sequencing of *BRCA1* exons 5, 20 and part of exon 11; *BRCA2* part of exons 10 and 11. | 1 variant identified in *BRCA2*:   - 1 patient with FHx OC |
| Riahi A, Kharrat M, Ghourabi ME, Khomsi F, Gamoudi A, Lariani I, et al. 2014. [57] | Tunisia | Evaluated 48 Tunisian women with early onset and familial BC and/or OC recruited from the Salah Azaiz Cancer Institute | *BRCA1* and *BRCA2* sequencing (Including exons and intron boundaries) | 1 variant identified in BRCA1:   - 1 patient with FHx OC |
| **Study** | **Country study conducted in** | **Setting/context** | **Genetic Test performed** | **Summary of main results** |
| Troudi W, Uhrhammer N, Sibille C, Dahan C, Mahfoudh W, Bouchlaka Souissi C, et al. 2007. [58] | Tunisia | Investigated the prevalence of *BRCA1* and *BRCA2* mutations in Tunisian BC patients with affected relatives in Tunisia | *BRCA1* and *BRCA2* sequencing | 4 variants identified in *BRCA1*:   - 3 patients with BC/OC - 1 patient with FHx BC/OC   1 variant identified in *BRCA2:*   - 1 patient with FHx BC/OC and OC |
| Pegoraro RJ, Moodley M, Rom L, Chetty R, Moodley J. 2003. [30] | South Africa | Blood samples were obtained from 45 Black South African women with OC presenting to the King Edward VIII Hospital, Durban, KwaZulu Natal. | Targeted mutation testing: (185delAG, 5382insC and 6174delT). | No variants identified |
| van der Merwe NC, Oosthuizen J, Theron M, Chong G, Foulkes WD. 2020. [59] | South Africa | Evaluated 744 patients with BC and/or OC for *BRCA1* or *BRCA2* large genomic rearrangements.  Zimbabwean patient included in cohort. | Large rearrangements in *BRCA1* and *BRCA2* using NGS/MLPA | 1 variant identified in *BRCA1*:   - 1 patient with FHx OC |
| Quiles F, Teulé À, Martinussen Tandstad N, Feliubadaló L, Tornero E, Del Valle J, et al. 2016. [40] | Spain and Norway | 11 individuals from five unrelated families of Moroccan origin in Spain and Norway were screened for *BRCA1* and *BRCA2* mutations. Families recruited from the Hereditary Cancer Program at the Catalan Institute of Oncology in Barcelona, Catalonia, Spain and Oslo University Hospital (Norway). | *BRCA1* and *BRCA2* sequencing | 2 variants identified in *BRCA1:*   - 1 patient with OC and FHx OC - 1 patient with FHx OC |
| **Study** | **Country study conducted in** | **Setting/context** | **Genetic Test performed** | **Summary of main results** |
| Broome CW. 2002. [60] | USA | 74 high risk African American breast cancer patients screened for BRCA2 mutations.  Washington DC, Howard University | *BRCA2* sequencing (coding regions and flanking intronic regions) | 2 variants identified in BRCA2:   - 1 BC patient with FHx OC - 1 OC patient with FHx OC |
| Churpek JE, Walsh T, Zheng Y, Moton Z, Thornton AM, Lee MK, et al. 2014. [61] | USA | Evaluated 289 patients who self-identified as African American with BC and high risk of having hereditary breast cancer syndrome recruited from The University of Chicago Medicine Breast Program and the Cancer Risk Clinic between 1993 and July 2013 | Panel testing: BRCA1, BRCA2, PALB2, CHEK2, ATM, BARD1, RAD51C, RAD51D, TP53, and PTEN | 9 variants identified in BRCA1:   - 3 patients with BC/OC - 6 patients with FHx OC   6 variants identified in BRCA2:   - 1 patient with BC/OC - 5 patients with FHx of OC |
| Gao Q, Neuhausen S, Cummings S, Luce M, Olopade OI. 1997. [42] | USA | Patients with a diagnosis of BC AND a 20% prior probability of being a BRCA1 carrier OR with a family history of at least two first-degree relatives with BC and/or OC <60 years of age were recruited through The University of Chicago Cancer Risk Clinic.  Cohort included African American patients | BRCA1 sequencing (coding regions)  Haplotype analysis | 2 variants identified in BRCA1:   - 2 patients with FHx OC |
| **Study** | **Country study conducted in** | **Setting/context** | **Genetic Test performed** | **Summary of main results** |
| Hung J, Mims B, Lozano G, Strong L, Harvey C, Chen TTY, et al. 1999. [62] | USA | Two apparently unrelated large African American families were studied. The first family BC54 was ascertained as part of a study of early onset and familial BC. The second family SARC-36, was ascertained from a series of childhood sarcoma patients. | TP53 sequencing | 2 variants identified in TP53:   - 2 patients with FHx BC/OC |
| Kanaan Y, Kpenu E, Utley K, Adams-Campbell L, Dunston GM, Brody L, et al. 2003. [63] | USA | 74 independent families with BC and/or OC at elevated risk of germline mutations in BRCA2 were recruited from Howard University Hospital and assessed for BRCA2 mutations.  African American patients were included in the cohort. | BRCA2 sequencing (coding regions and flanking introns) | 2 variants identified in BRCA2:   - 2 patients with FHx of OC |
| Kurian AW, Ward KC, Howlader N, Deapen D, Hamilton AS, Mariotto A, et al. 2019. [64] | USA | Study included BC and OC patients from the Georgia Cancer Registry and the California Cancer Registry  African American patients were included in the cohort. | Gene panel testing BRCA1, BRCA2, BRIP1, EPCAM, MLH1, MSH2, MSH6, PMS2, RAD51C, RAD51D, and STK11 | 7 variants identified in BRCA1:   - 7 patients with OC |
| Lynce F, Smith KL, Stein J, DeMarco T, Wang Y, Wang H, et al. 2015. [37] | USA | Retrospective study to determine the prevalence of deleterious BRCA1/2 mutations in African American women from two genetic counselling clinics at hospitals in Washington, DC | BRCA1 and BRCA2 sequencing | 2 variants identified in BRCA1:   - 2 patients with OC |
| Mefford HC, Baumbach L, Panguluri RCK, Whitfield-Broome C, Szabo C, Smith S, et al. 1999. [38] | USA | Genotyping of BC and OC families believed to be of West African ancestry | Targeted testing (Founder mutation: *BRCA1* 943ins10)  Haplotype analysis | 3 variants identified in *BRCA1*:   - 1 patient with BC/OC - 2 patients with FHx OC |
| **Study** | **Country study conducted in** | **Setting/context** | **Genetic Test performed** | **Summary of main results** |
| Nanda R, Schumm LP, Cummings S, Fackenthal JD, Sveen L, Ademuyiwa F, et al. 2005. [43] | USA | Comparative analysis of families (European, Ashkenazi Jewish, African American, Hispanic, Asian) with 2 or more cases of BC and/or OC among first- and second-degree relatives recruited from the Cancer Risk Clinic at the University of Chicago, Mayo Clinic, Rush University Medical Centre, and the University of California-San Francisco. | *BRCA1* and *BRCA2* sequencing | 4 variants identified in *BRCA1*:  4 patients with FHx OC |
| Norquist BM, Harrell MI, Brady MF, Walsh T, Lee MK, Gulsuner S, et al. 2016. [27] | USA | 1915 woman with OC from the University of Washington (UW) gynecologic tissue bank and from Gynecologic Oncology Group (GOG) phase III clinical trials.  African American patients were included in the cohort. | NGS panel ATM, BARD1, BRCA1, BRCA2, BRIP1, CHEK2, FAM175A, FANCP, MLH1, MSH2, MSH6, MRE11A, NBN, PALB2, PMS2, PTEN, RAD50, RAD51C, RAD51D, and TP53 | 1 variant identified in PALB2:   - 1 patient with OC   1 variant identified in PMS2:   - 1 patient with OC |
| Pal T, Vadaparampil S, Betts J, Miree C, Li S, Narod SA. 2008. [65] | USA | Study investigated BRCA1 and BRCA2 mutations among 51 African American BC patients with a personal or family history suggestive of hereditary predisposition to BC. Recruitment included patients from the Moffitt Cancer Centre Breast Program, the Florida State Cancer Registry and other referrals. | BRCA1 exon 11; BRCA2 exon 10 and 11 | 1 variant identified in BRCA1:   - 1 patient with FHx OC |
| Panguluri RC, Brody LC, Modali R, Utley K, Adams-Campbell L, Day AA, et al. 1999. [39] | USA | BRCA1 was analysed for germline mutations in 45 African American families at high-risk for hereditary breast cancer recruited from Howard University Cancer Center | BRCA1 sequencing (coding regions and flanking intronic region) | 1 variant identified in BRCA1:   - 1 patient with BC/OC |
| **Study** | **Country study conducted in** | **Setting/context** | **Genetic Test performed** | **Summary of main results** |
| Safra T, Lai WC, Borgato L, Nicoletto MO, Berman T, Reich E, et al. 2013. [28] | USA, Israel, Italy | Retrospectively reviewed BRCA testing in patients diagnosed with EOC from New York University Cancer Institute (USA), Tel Aviv Sourasky Medical Center and (Israel) Istituto Oncologico Veneto (Italy)  African American patients were included in the cohort. | TASMC: Targeted mutation analysis BRCA1 Y978, 185delAG, A1708, and 5382insC) and BRCA2 exon 11 (6174delT),  NYU and IOV: BRCA1 and BRCA2 sequencing | 1 variant identified in BRCA1:   - 1 patient with OC |
| Sia TY, Maio A, Kemel YM, Arora KS, Gordhandas SB, Kahn RM, et al. 2023. [29] | USA | Patients with confirmed EOC underwent clinical tumor-normal sequencing from 2015, to 2020, inclusive of germline analysis of ≥76 genes (New York)  African American patients were included in the cohort. | Panel testing ≥76 genes | 6 variants identified in BRCA1:   - 6 patients with OC   1 variant identified in BRCA2, PMS2, BARD1, CHEK2, NTHL1 in 5 patients with OC |
