# Supplementary material for: Hereditary ovarian cancer in women with African ancestry: a scoping review
Source: Fam Cancer. 2026 Jan 31;25(1):16. doi: 10.1007/s10689-026-00530-x (PMC12860814; doi:10.1007/s10689-026-00530-x)

**Online resource 3**

**Disease-causing variants identified in patients and families with a history of ovarian cancer**


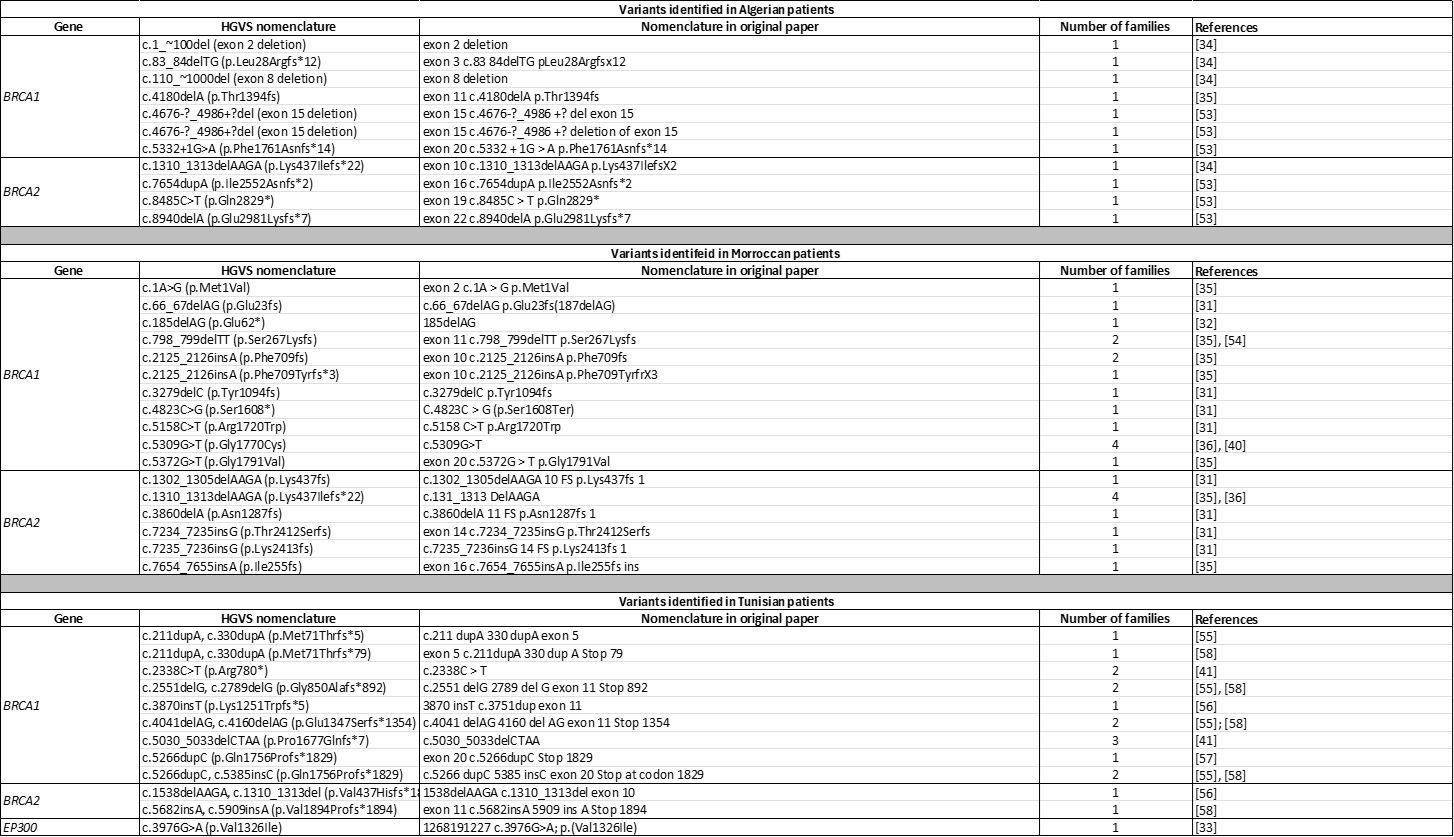


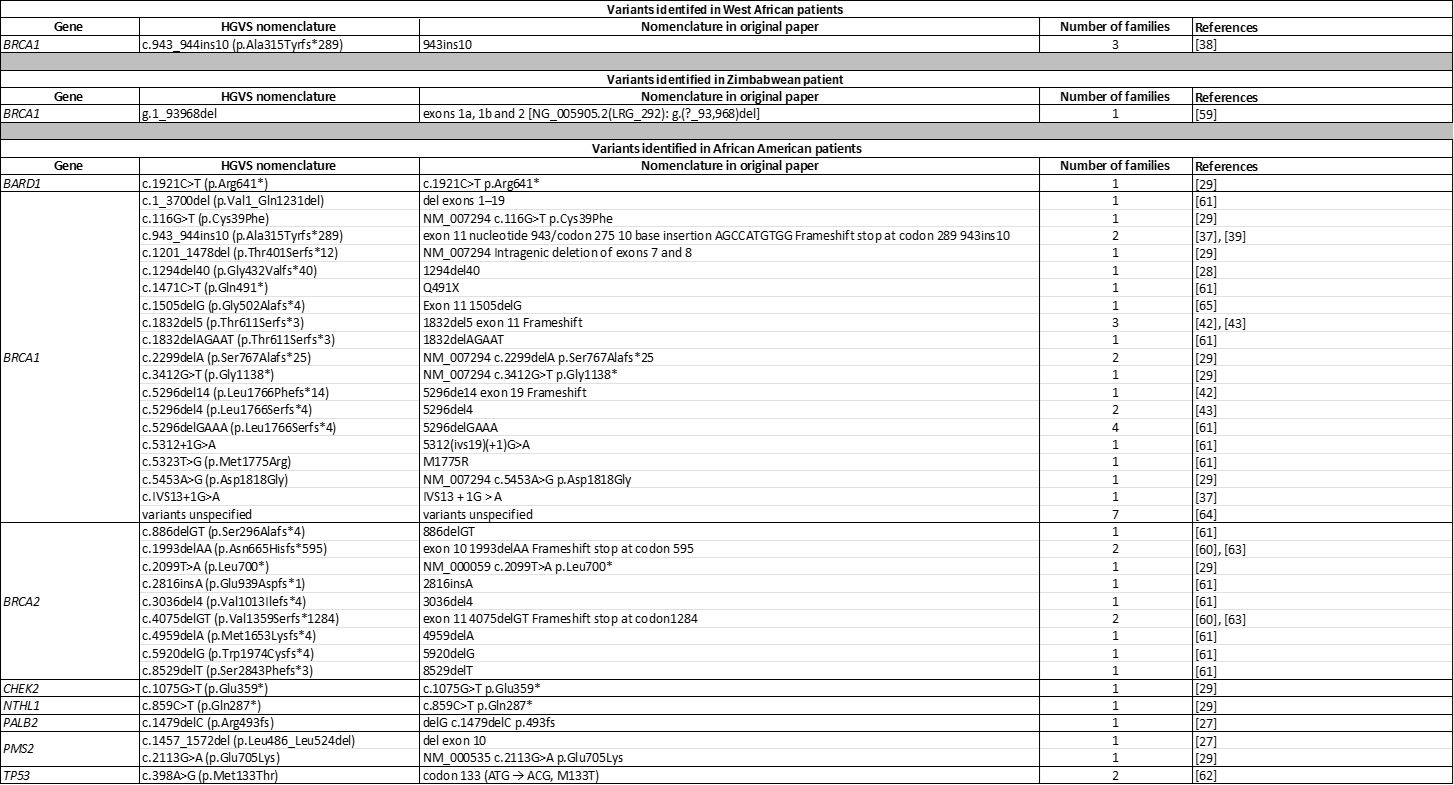

Supplement: Supplementary file 3 — Supplementary Material 3 [file 10689_2026_530_MOESM3_ESM.docx]
